# Determinants of Left Ventricular Diastolic Dysfunction in Normotensive Patients With Type 2 Diabetes Mellitus: A Tertiary Hospital-Based Study in Ghana

**DOI:** 10.7759/cureus.104317

**Published:** 2026-02-26

**Authors:** Forster N Fokuoh, Abdul-Subulr Yakubu, Franscis Agyekum, Aba Folson, Rafiq Okine, Evans A Asamoah, John Kpodonu, Eugene Amable, Alfred Doku

**Affiliations:** 1 Internal Medicine, University of Ghana Medical Centre, Accra, GHA; 2 Internal Medicine, Tamale Teaching Hospital, Tamale, GHA; 3 Internal Medicine, University of Ghana Medical School, Accra, GHA; 4 Internal Medicine, University of Health and Allied Sciences, Ho, GHA; 5 Public Health, World Health Organization, Geneva, CHE; 6 Molecular Medicine, AG-Amuasi Group, Kumasi Centre for Collaborative Research in Tropical Medicine, Kumasi, GHA

**Keywords:** complications of diabetes, diabetic cardiomyopathy, ghana, left ventricular diastolic dysfunction, type 2 diabetes

## Abstract

Background

Diabetes mellitus is a growing global health challenge, with significant cardiovascular complications among affected populations. Left ventricular diastolic dysfunction (LVDD) represents an early manifestation of diabetic cardiomyopathy and predicts adverse outcomes. Data on LVDD in normotensive patients with type 2 diabetes mellitus (T2DM) in Ghana are limited. This study aimed to estimate the prevalence and identify determinants of LVDD among normotensive T2DM patients presenting to the Korle-Bu Teaching Hospital in Ghana.

Methodology

This was a descriptive cross-sectional study of randomly selected normotensive T2DM patients attending the National Diabetes Management and Research Centre at the Korle-Bu Teaching Hospital between January 2016 and December 2016. Transthoracic echocardiography was performed, and left ventricular diastolic function was assessed and graded based on pulsed Doppler of the mitral inflow velocities and tissue Doppler imaging of the mitral annuli.

Results

Three hundred and sixty-five participants were recruited, of whom 254 (69.6%) were female. The mean age of the participants was 50.7 ± 8.2 years. Of the 365 participants enrolled, 356 (97.5%) had complete echocardiographic data; nine participants with inconclusive findings were excluded from the analysis. One hundred and fifty-two participants (42.7%, 95%CI 37.5%-47.9%) had evidence of LVDD, comprising mostly grade 1 LVDD (96.1%). Older age (aOR 1.160, 95%CI 1.108-1.214, *p*<0.001), increased septal wall thickness (aOR 7.628, 95%CI 1.160-50.176, *p*=0.035), and a higher heart rate (aOR 1.037, 95%CI 1.007-1.067, *p*=0.016) were independently associated with the presence of LVDD. Participants with LVDD were more likely to be symptomatic.

Conclusion

Mild LVDD was common among normotensive Ghanaian patients with T2DM, with age, heart rate, and cardiac remodeling associated with its presence. These findings underscore the potential value of echocardiographic screening in this population, while longitudinal studies are needed to establish prognostic significance.

## Introduction

The prevalence of diabetes mellitus (DM) is on the ascendency worldwide. According to the World Health Organization, 24.6 million adults are living with diabetes in Africa, with the figure projected to rise by 142% to 60 million by 2050 [1]. It is estimated that the prevalence of DM in Ghana is about 6.8% [2].

Cardiovascular complications remain the leading cause of morbidity and mortality among individuals with type 2 diabetes mellitus (T2DM) [3]. Beyond traditional risk factors, including hypertension, T2DM induces distinct alterations in the myocardium that contribute to left ventricular diastolic dysfunction (LVDD). LVDD is widely regarded as an early marker of diabetic cardiomyopathy, preceding the onset of symptomatic heart failure [4-6]. Early identification and management of LVDD may therefore slow or prevent progression to overt heart failure [7]. Echocardiographic screening offers a valuable opportunity to detect this preclinical stage, potentially unveiling a large group of patients in whom timely intervention could alter disease trajectory [7].

Globally, LVDD is highly prevalent among individuals with T2DM, with approximately half of patients showing evidence of LVDD [8]. There is a paucity of data on the predictors of LVDD among patients with T2DM in Ghana and across sub‑Saharan Africa, with available studies suggesting a prevalence exceeding 60% [9]. Given the limited data on LVDD predictors in sub‑Saharan Africa, particularly among normotensive patients with T2DM, and to minimize confounding from hypertension, a well‑established determinant of LVDD, we sought to determine the prevalence and associations of LVDD in normotensive T2DM patients presenting to the National Diabetes Management and Research Centre at the Korle‑Bu Teaching Hospital in Ghana. The primary objective of this study was to estimate the prevalence of LVDD among normotensive patients with T2DM. Secondary objectives included identifying associations of LVDD with key exposures of interest, such as age, diabetes duration and echocardiographic structural parameters. 

## Materials and methods

Study design and setting

This was a descriptive cross-sectional study of normotensive patients with T2DM attending the National Diabetes Management and Research Centre, Korle-Bu Teaching Hospital, from January 2016 to December 2016. Korle-Bu Teaching Hospital is a tertiary referral hospital in Ghana with 16 specialized units, including the study site. The Centre runs daily clinics from Monday to Friday with an average daily attendance of about 80 patients. Patients can walk into the Diabetes Centre, or they may be referred from hospitals and clinics across the country. The Centre, headed by a Diabetologist, is a unit under the Department of Medicine and Therapeutics.

Study population

Inclusion criteria were age 35 to 65 years with a diagnosis of T2DM and normal blood pressure. The lower age limit of 35 years was chosen to remove the likelihood of including type 1 diabetics, whilst an upper age cut-off of 65 years was chosen to limit the influence of age on diastolic function. We excluded patients with T2DM and controlled hypertension, patients with type 1 diabetes, those with valvular heart disease or symptomatic heart failure and those with a history of myocardial infarction or congenital heart disease.

A minimum sample size of 365 participants was required based on the prevalence of LVDD from previous studies in the sub-region, after accounting for a non-response rate of 5% [9]. Normotensive patients with T2DM who consented were consecutively sampled during clinic visits until a group of 10 was reached. Of the 10 participants who met the inclusion criteria, six were randomly selected through simple balloting. Specifically, each participant was assigned a unique identification number. These numbers were written on identical slips of paper, folded to ensure concealment, and placed into an opaque container. Six slips were then drawn at random without replacement to determine participant selection. This process was repeated until the target sample size was achieved. 

Data collection

A thorough history and physical examination were performed to identify comorbidities and any exclusion criteria. Glycated hemoglobin, fasting blood glucose, and fasting lipid profiles were assessed at the central laboratory of Korle-Bu Teaching Hospital. Diabetes mellitus was diagnosed based on the presence of symptoms of diabetes and a fasting plasma glucose > 7 mmol/l or 2-hour postprandial glucose greater than or equal to 11 mmol/l or known T2DM patients who were on oral hypoglycemic agents or who, during treatment, came to require insulin for control of plasma glucose. The duration of T2DM was determined based on the patient's self‑reported time since diagnosis.

Blood pressure was measured in a quiet environment with an automated Omron 761 blood pressure apparatus (Omron Healthcare Co., Ltd., Kyoto, Japan) with the patient in a sitting position with the feet flat on the ground after five minutes of rest. Three consecutive blood pressure readings were taken in the arm with the higher pressure, and the reported value was the average of the second and third readings. Hypertension was defined as an office systolic blood pressure ≥140 mmHg and or diastolic blood pressure ≥ 90 mmHg or if taking blood pressure-lowering medication [10]. Participants wearing light clothes without shoes stood on a level placed weighing scale (Salter 200 Academy professional mechanical, Salter Housewares Ltd, Kent, United Kingdom) for the weights. Height measurements were determined using a stadiometer (Seca 213 stadiometer, Seca GmbH & Co. KG, Hamburg, Germany).

A 12-lead electrocardiogram (ECG) was performed using standard techniques and settings (Schiller AT 10 plus ECG machine, Schiller AG, Baar, Switzerland). Trans-thoracic echocardiography evaluation was performed using a Toshiba Aplio 300 ultrasound system (Toshiba Medical Systems, Otawara, Japan) equipped with a 3.5 MHz phased-array probe according to the recommendations of the American Society of Echocardiography (ASE) [11-13]. All echocardiographic studies were performed by a certified cardiology fellow under the supervision of an experienced consultant cardiologist. To ensure consistency and minimize interobserver variability, all measurements were systematically verified by a third senior cardiologist. Participants with evidence of left ventricular wall motion abnormalities and poor left ventricular ejection fraction (EF< 50%) were excluded from the data analysis. Pulsed Doppler of the mitral inflow velocities (early (E-wave) and late (A-wave)) was performed, and their ratio (E/A) was calculated. The deceleration time (DT) of the E-wave was determined. Left ventricular myocardial tissue velocities were evaluated by pulsed tissue Doppler imaging (TDI). The peak early diastolic (e’) velocities of the lateral and septal mitral annulus were measured by TDI and averaged. The ratio between the E and the e’ wave (E/e’) was calculated as a pre-load independent index of LV filling pressures.

Diastolic function was classified using criteria adapted from the 2009 ASE guidelines, which were the prevailing recommendations at the start of data collection [13]. Grade 1 LVDD was defined as an E/A < 0.8, a DT > 200 ms and an average E/e’ ≤ 8. Grade 2 LVDD was present if the E/A ratio was between 0.8 and 2, with a DT of 160-200ms and an average E/e’ ≥ 13, whilst grade 3 LVDD was defined as an E/A > 2, a DT < 160ms and an average E/e’ >13.

Statistical analysis

Data were analyzed using IBM SPSS Statistics for Windows, Version 22 (Released 2013; IBM Corp., Armonk, New York, United States). Categorical variables were presented as counts and percentages, whilst continuous variables were presented as means and standard deviations. The relationship between LVDD and categorical variables was tested using the Chi-square test, whilst the means of continuous variables were compared using the independent sample Student's t-test. Univariate logistic regression analysis was performed to identify the predictors of LVDD. Variables that were significantly associated with LVDD in the univariate analysis were entered into a multiple stepwise (entry level of p < 0.1) logistic regression model. A p-value less than 0.05 was considered statistically significant. All continuous variables included in the logistic regression model were entered into a linear regression, and collinearity diagnostics were requested. Variance inflation factor (VIF) and tolerance values were examined, with VIF > 5 or tolerance < 0.2 considered indicative of potential collinearity.

Missing values were excluded from the analysis without imputation. Of the 365 participants enrolled, 356 had complete data for diastolic function classification. Nine participants with inconclusive echocardiographic data, due to poor image quality or borderline values that precluded classification according to our criteria, were excluded from the analysis.

Ethical considerations

The study complied with the principles outlined in the Declaration of Helsinki on the ethical principles for medical research involving human subjects. Ethical approval for this study was obtained from the Ethical and Protocol Review Committee of the Korle-Bu Teaching Hospital (Approval number: KBTH-IRB/0009/2016).

## Results

A total of 365 participants with a mean age of 50.7±8.2 years were recruited for the study, comprising 254 (69.6%) female participants (data not shown). The ages of female participants (50.3±8.0 years) and male participants (51.6±8.7 years) did not differ significantly (t(363) = 1.363, p = 0.174).

The average duration of diabetes was 8.1 ± 5.5 years. Metformin and a sulfonylurea combination was the commonest oral hypoglycemic therapy used. About a third of the participants (32.1%) were receiving insulin as part of their treatment regimen. Statin therapy was employed in 142 (38.9%) participants.

Out of the 365 recruited participants, 356 (97.5%) had their LV diastolic function assessed. One hundred and fifty-two participants (42.7%, 95%CI 37.5%-47.9%) had evidence of LVDD, of which 146 (96.1%) had grade 1 LVDD, and 6 (3.9%) had grade 2 LVDD. Participants with LVDD were older, had T2DM for a significantly longer duration and were more likely to report easy fatigability than those without LVDD (Table 1). There was no sex or BMI difference between those with or without LVDD, though female participants had a higher BMI (28.6 vs 24.7, t (359) =6.877, p<0.001) and waist circumference (95.1 vs 89.7, t (351) =3.726, p<0.001) compared with men. Table 1 summarizes the clinical and biochemical parameters of participants with and without LVDD. There were no significant differences in biochemical parameters between those with and without LVDD.

**Table 1:** Clinical and biochemical characteristics of participants BMI, Body Mass Index; BSA, Body Surface Area; FBG, Fasting Blood Glucose; HBA1C, Hemoglobin A1c; HDL, High-Density Lipoprotein; LDL, Low-Density Lipoprotein; Met, Metformin; SU, Sulfonylurea; TDZ, Thiazolidinediones; TG, Triglyceride “Others” refers to other medication class combinations. Values are presented counts (%) or means ± standard deviation.  Categorical and continuous variables were compared using the Chi-squared and independent sample student t-tests, respectively.  Values with * are significant at the 0.05 α level.

Variables	LVDD Status		Total (n = 356)	Test statistic	df	p-value
	No LVDD (n = 204)	LVDD (n = 152)				
Sex				X^2^=2.609	1	0.132
Female	134	112	246			
Male	70	40	110			
Age of participants (years)	47.8±7.4	54.9±7.1	50.7±8.2	t=9.127	354	<0.001^*^
Anti-diabetic agents				X^2^=9.312	5	0.097
Metformin	32	15	47			
Mixtard insulin	18	10	28			
Mixtard insulin + Metformin	43	46	89			
Met + SU	76	52	128			
Met + SU + TDZ	15	4	19			
Others	9	7	16			
Statin therapy				X^2^=2.270	2	0.321
Atorvastatin	72	43	115			
Rosuvastatin	9	10	19			
Simvastatin	6	2	8			
Duration of diabetes (years)	7.3±5.6	9.1±5.1	8.1±5.5	t=2.932	342	0.004*
Hemodynamic measurements						
Average SBP (mmHg)	116.8 ± 11.5	121.1 ± 11.4	119.8±11.4	t=3.396	345	0.001*
Average DBP (mmHg)	72.4 ± 9.9	72.7 ± 7.3	72.7 ± 7.4	t=0.341	346	0.733
Heart rate	71.4 ± 12.9	75.1 ± 11.1	73.2 ± 12.1	t=2.322	229	0.021*
Physical measurements						
BMI (kg/m^2^)	27.3 ± 5.0	27.6 ± 5.8	27.4±5.3	t=0.766	350	0.444
Waist circumference (cm)	93.7 ± 11.4	94.1 ± 13.6	93.6±12.6	t=0.299	342	0.765
Waist/hip ratio	0.94 ± 0.10	0.96 ± 0.17	0.95±0.13	t=1.211	341	0.227
BSA (m^2^)	1.78 ± 0.16	1.75 ± 0.19	1.77 ± 0.18	t=1.744	350	0.082
Biochemical measurements						
HBA1C (%)	7.78 ± 2.13	7.92 ± 2.53	7.83±2.30	t=0.906	312	0.569
Total cholesterol (mmol)	4.97 ± 1.26	4.99 ± 1.26	4.98±1.26	t=0.146	310	0.884
LDL cholesterol(mmol)	3.21 ± 1.13	3.26 ± 1.40	3.23±1.13	t=0.388	312	0.699
HDL-C (mmol)	1.40 ± 0.48	1.39 ± 0.44	1.41±0.46	t=0.084	312	0.933
TG (mmol)	1.08 ± 0.58	1.12 ± 0.57	1.10±0.57	t=0.559	310	0.576
FBG (mmol)	8.69 ± 3.12	8.91 ± 3.38	8.78±3.24	t=0.622	341	0.535
Symptoms						
Easy fatigability	25	41	66	X^2^=14.758	1	<0.001^*^
Pedal edema	3	7	10	X^2^=3.574	1	0.099
Palpitations	16	19	35	X^2^=2.827	1	0.093
Ever smoked	2	4	6	X^2^=1.708	1	0.191
Alcohol consumption	17	14	31	X^2^=0.228	1	0.633

The echocardiographic findings of the participants are summarized in Table 2. The septal wall thickness, left ventricular mass index, left ventricular ejection fraction, aortic root diameter, and heart rate were significantly higher among participants with LVDD than those without LVDD.

**Table 2:** Comparison of echocardiographic measurements according to diastolic function EDD, End-Diastolic Diameter; LV, Left Ventricle Values are presented as means ± standard deviation and compared using the independent sample student t-tests.  Values with * are significant at the 0.05 α level. The echocardiographic measurements were made with the Toshiba Aplio 300 ultrasound system equipped with a 3.5 MHz phased-array probe [11].

Variable (Mean ± SD)	LVDD		Test statistic	df	p-value
	NO LVDD (n=204)	LVDD (n=152)			
Septal wall thickness (cm)	1.01 ± 0.15	1.08 ± 0.19	t=3.994	353	< 0.001*
LV posterior wall (cm)	0.94 ± 0.16	0.96 ± 0.17	t=1.071	353	0.285
Left ventricle EDD (cm)	4.41 ± 0.39	4.36 ± 0.57	t=0.908	354	0.364
Aortic root diameter (cm)	2.95 ± 0.28	3.08 ± 0.31	t=4.420	353	< 0.001*
LV mass index (g/m^A^)	83.51 ± 17.47	89.03 ± 24.44	t=2.469	350	0.014*
Deceleration time (s)	0.21 ± 0.04	0.21 ± 0.04	t=0.021	354	0.983
Ejection fraction (%)	56.23 ± 5.75	54.91 ± 6.32	t=2.025	347	0.044*
Left atrial area (cm2)	14.87 ± 2.48	14.58 ± 4.74	t=0.686	315	0.487
Heart rate per min	71.40 ± 12.90	75.08 ± 11.14	t=2.322	229	0.021*

A stepwise logistic regression analysis was conducted to ascertain the effects of various demographic, clinical and echocardiographic characteristics on the likelihood that the participants would have LVDD. Variables included in the multivariable model included age, diabetes duration, systolic blood pressure, medications, septal wall thickness, left ventricular mass index, heart rate, aortic root diameter, left ventricular ejection fraction and symptoms (easy fatigability, pedal edema, palpitations). The final model was statistically significant (X2(3)=62.647, p<0.001) and explained 33.3% of the variance in LVDD and correctly classified 73.4% of the cases (Table 3). The Hosmer-Lemeshow goodness‑of‑fit test indicated adequate model fit (χ² = 4.098, df = 8, p = 0.848). All predictors had VIF values < 2 and tolerance values > 0.5, indicating no significant collinearity.

**Table 3:** Independent predictors of LVDD aOR, Adjusted Odds Ratio; CI, Confidence Interval p-values with * are significant at the 0.05 α level. Nagelkerke R2 =0.333, p<0.001 For continuous variables, the odds ratio represents the factor by which the odds of the outcome change for a unit increase in the continuous predictor. The echocardiographic measurements were made with the Toshiba Aplio 300 ultrasound system equipped with a 3.5 MHz phased-array probe [11].

Variable	aOR	95% CI	p-value
Age	1.160	1.108-1.214	<0.001
Septal wall thickness	7.628	1.160-50.176	0.035
Heart rate	1.037	1.007-1.067	0.016

## Discussion

Data on the association between T2DM and LVDD in native African populations are limited, with most available studies involving relatively small cohorts [9,14,15]. This study, one of the largest to date, sought to identify predictors of LVDD among normotensive T2DM patients presenting to a tertiary facility in Ghana. Nearly half of the participants (42.7%) had evidence of LVDD, though this prevalence remains lower than previous estimates reported in the subregion [9,14,15]. Older age, increased septal wall thickness, and a higher heart rate were significantly associated with the presence of LVDD. Consistent with other studies, the majority of cases comprised grade I dysfunction. Most participants were asymptomatic, whereas patients with higher grades would likely have been symptomatic and diagnosed with heart failure. This is important because identifying dysfunction at an earlier stage may allow tailored treatment to prevent progression.

The female predominance of the sample is reflective of the patient population at the study site [16]. There was no gender difference in the prevalence of LVDD in this study. However, women were more likely to be overweight, consistent with the literature on the obesity rates of women in this part of Ghana [17]. A female predisposition to LVDD in T2DM patients has previously been reported and attributed to differences in molecular mechanisms such as oxidative stress and sex differences in calcium handling that may increase cardiovascular risk in women with T2DM [18,19]. The absence of the protective effect of estrogen on postmenopausal women increases their risk of cardiovascular disease [20].

Age was an independent predictor of LVDD in this study. The changes that occur in the cardiovascular system with increasing age, such as increased cross-linking of collagen, an increase in smooth muscle content and a significant loss of elastic fibers, seem to be markedly accelerated by DM, predisposing to LVDD [21]. These changes cause a decrease in the compliance of the left ventricle and, hence, the development of LVDD. Insulin resistance and hyperglycemia in patients with T2DM are believed to lead to structural and functional changes in the cardiomyocytes that can result in a steady impairment of left ventricular diastolic function [22]. However, indices of hyperglycemic control, such as HbA1c and fasting glucose levels, were not associated with the presence of LVDD in this study. Reports of the association between LVDD and the HbA1c have produced mixed results [23-25]. This suggests that structural and hemodynamic factors may play a dominant role in LVDD pathophysiology in this population, emphasizing the need for mechanistic studies to clarify these pathways. Given that this study assessed HbA1c at a single time point, future investigations incorporating measures of long‑term variability would provide a more comprehensive evaluation of the impact of glycemic control on LVDD.

Longer duration of diabetes has been associated with the presence of LVDD [26,27]. Possible mechanisms include increased risk of atherosclerotic coronary artery disease and diabetic cardiomyopathy resulting from cardiac microvascular dysfunction. Structural changes in the left ventricle resulting from the deposition of advanced glycosylated end products can lead to an increased left ventricular mass and the onset of LVDD, as well as elevated left ventricular end-diastolic pressure [28,29]. In the univariate analysis, participants with LVDD had T2DM for a significantly longer duration than those without LVDD (9.1 vs 7.3 years, p=0.003). However, diabetes duration was not significant in the multivariable analysis. Residual confounding with age cannot be excluded, as diabetes duration became an independent predictor when the analysis was repeated without age in the model.

This study further found that LVDD was associated with a higher resting heart rate, which may reflect autonomic dysfunction in these patients, a phenomenon that is associated with poor cardiovascular outcomes [30]. Long-standing increase in heart rate may increase cardiac oxygen requirements, leading to left ventricular remodeling and later LVDD by shortening the duration of diastole. Several echocardiographic structural variables were associated with the presence of LVDD in the univariate analysis; however, only septal wall thickness remained independently associated with LVDD in the multivariate analysis.

Although this study recruited normotensive patients, it was observed that participants with LVDD had a significantly higher average systolic blood pressure than those without LVDD. This observation that higher systolic blood pressure values within the normotensive range were associated with LVDD suggests that even subtle elevations may have pathophysiological implications, warranting closer attention to treatment targets. Further studies are needed to determine if diabetic patients with LVDD would benefit from blood pressure-lowering medication at a lower threshold than is currently recommended in the guidelines.

In addition to its single-center design, the study had some important limitations. As a tertiary diabetes center, our participants are likely to represent more complex cases, which may limit the generalizability of findings to broader populations. Information about coronary anatomy was not available to exclude epicardial CAD as the underlying cause of LVDD, and masked hypertension could not be excluded, as out-of-office blood pressure monitoring was not performed. The cross‑sectional design further limits causal inference and raises the possibility of reverse causation. Additionally, diastolic function was assessed mainly with pulsed and tissue Doppler characteristics. Our decision to use 35 years as the lower age limit was applied as a pragmatic safeguard to reduce misclassification, while recognizing that some individuals under 35 may indeed have T2DM. Similarly, excluding older adults to minimize the confounding influence of age on diastolic function may reduce clinical applicability, given the rise in LVDD with age. Finally, standardized effect sizes were not calculated in our dataset, and multiple comparisons were performed, increasing the risk of type I error. Therefore, statistical significance should be interpreted with caution, and findings should be considered in light of their clinical relevance rather than p‑values alone. 

## Conclusions

Mild LVDD was present in almost half of these normotensive Ghanaian patients with T2DM. Advanced age, higher resting heart rate, and evidence of cardiac remodeling were independently associated with LVDD. These findings highlight the potential clinical value of incorporating echocardiographic screening in this population, particularly for identifying subclinical dysfunction. While the prognostic significance of predominantly grade I LVDD remains uncertain, longitudinal studies are needed to clarify its trajectory and determine whether early identification can inform risk stratification and management strategies.
